# Wireless pressure insoles for measuring ground reaction forces and trajectories of the centre of pressure during functional activities

**DOI:** 10.1038/s41598-023-41622-3

**Published:** 2023-09-11

**Authors:** T. Cudejko, K. Button, M. Al-Amri

**Affiliations:** 1 Department of Sport, Exercise and Rehabilitation, Northumbria University, Newcastle Upon Tyne, NE1 8ST UK; 2 School of Healthcare Sciences, College of Biomedical and Life Sciences, Cardiff University, Cardiff, CF14 4EP UK

**Keywords:** Biomedical engineering, Rehabilitation

## Abstract

Wireless pressure insoles may enable the assessment of movement biomechanics in a real-world setting, and thus play an important role in the recommendation of clinical management, but they are not yet a gold standard due to the unknown accuracy and reliability with respect to different functional activities. Here, we compare novel wireless pressure insoles with force plates and examine the test–retest reliability of the insoles for measuring vertical ground reaction forces (vGRFs) and trajectories of the center of pressure (COP). In this observational study, healthy adults underwent two data collection sessions during one day. The Bland–Altman analysis was used to compare the outcomes measured with the two instruments during squats, jumps, and the sit-to-stand test. Test–retest reliability was assessed by the interclass correlation coefficient and the standard error of measurement for the outcomes during squats, jumps, walking, and stair ambulation. Trajectories of the COP in the anterior–posterior direction were comparable between the two systems during all activities. The insoles consistently measured shorter trajectories of the COP in the medial–lateral direction (except jumps) and lower vGRFs than the force plates. Test–retest reliability of the insoles was fair to high or excellent for all outcomes during all activities. In conclusion, the insoles provide reliable measures of vGRFs and trajectories of the COP during multiple functional activities in healthy adults. Although the insoles do not produce identical results to the force plate, the qualitative similarity and consistency between the two systems confirm the insoles can be used to measure these outcomes, based on the purpose and accuracy required.

## Introduction

Human movement analysis has a wide range of clinical applications. However, due to the pricey, cumbersome, and highly specialised equipment needed for these purposes, research investigations using movement analysis are typically restricted to academic research centres and small sample numbers. To remedy this situation, there has been a rise in research developing in-expensive and easy-to-use wearable technologies, such as inertial measurement units (IMUs)^1,2^. These systems, however, are restricted to measuring kinematic or spatio-temporal movement parameters. However, foot pressures, such as trajectories of the center of pressure (COP), or kinetics, such as ground reaction forces (GRF), are currently not possible to directly measure with these instruments, with the latter necessitating prediction using inverse dynamics-based methods (and additional equipment such as force/pressure plates) or the machine learning-based methods^3^.

Measurement of trajectories of the COP has been utilized in a wide range of clinical applications including assessment of postural stability in people after stroke^4^, with diabetic neuropathy^5^, or with Parkinson’s disease^6^, assessment of treatment after hip osteoarthritis^7^, or in prosthetic design^8^. The vertical component of GRFs (vGRFs) is frequently used in the evaluation of both normal and pathological movement as a means of understanding various loading parameters^9^, and has been shown utility in monitoring fracture healing^10^, assessing fall risk^11^, and in rehabilitation research^12^. Currently, vGRFs and trajectories of the COP are usually measured with force platforms, and pressure plates in the case of the latter. However, these instruments can only record either a single step or a few steps at a time and limit participants to a single environment, and therefore allow only a momentary view of the patient’s movement in a confined research environment. The relevance of the measurement results of such a reduced time frame for the real world is rather limited. Given that most disease processes are long-term, tools with continuous measuring capabilities are needed.

In the last decade, there has been a rise in research developing wearable technologies capable of overcoming these challenges, such as wireless pressure insoles. Such systems may enable the assessment of vGRFs and COP trajectories during functional activities in a real-world setting and offer higher efficiency, flexibility, and mobility than traditional laboratory-based, force and pressure plates. The justification of using a particular insole technology will depend on research and/or clinical purposes. Insoles with capacitive sensors for instance, may be subject to creep when exposed to prolonged periods of loading^13^, potentially making them less effective when assessing static tasks. Such devices may also be used due to their capacity to detect small changes in plantar pressure in response to orthotic or footwear intervention, when reducing the risk of foot ulceration in people with diabetes^14^. Commercial wireless insoles are increasingly available, to name a few: Moticon GmbH®, Medilogic®, eSHOE ®, F-Scan®, Pedar®. Recently, X4 Foot & Gait Measurement System® was launched by the XSENSOR. The system is commercially available and consists of a pair of wireless sensor insoles, with integrated internal storage. By releasing participants from the shortcomings of cables and additional devices for data storage, it may provide a new way of conducting human motion analysis.

The recent commercial developments of insole pressure measurement systems necessitated a comprehensive understanding of the accuracy and precision of such systems^15–19^. The current limitation of the research in this field is its restriction to assessing usually only walking. The task performed during a clinical assessment establishes the time frame, rate, and area of the pressure application in addition to the portion of the insole that is under pressure. Long-term static loading differs from cyclic dynamic loading in terms of the conditions under which it occurs, and these conditions call for various qualities from the insole systems. The intensity and length of these applied pressures affect the sensors' dynamic response and, consequently, the outcome variables. Research is scarce regarding how well insole systems perform during activities other than walking, and in particular, their performance is unknown in more challenging activities such as squats, jumping or stair ambulation, which may be performed at variable speeds and may sustain a substantial load through both feet throughout the duration of activity.

Information on clinimetric properties of the X4 Foot & Gait Measurement System in a healthy population is needed to determine if the system is suitable for future validation in patient populations. Recently, Parker et al., used a pressurised bladder to apply an even load to the X4 insole surface at a range of pressures. They demonstrated high validity and reliability of the insoles for measuring contact area, mean pressure and peak pressure^14^. To our knowledge, a thorough analysis of the validity and repeatability of this insole system in humans is lacking. Therefore, the objective of the study was to compare the X4 wireless pressure insoles with force plates and examine the test–retest reliability of the insoles for measuring the vGRFs and COP parameters during multiple functional activities in healthy adults.

## Methods

### Study design

In this observational study individuals undertook three data collection sessions during one day, as part of a larger study^2^. To achieve the current research objectives, here, we present data collected in the first and the second data collection sessions. All assessments were conducted at the School of Healthcare Sciences at Cardiff University, in the period between September 2021 and December 2021. We followed the Guidelines for Reporting Reliability and Agreement Studies^20^. The study was approved by the School of Healthcare Sciences Research Ethics Committee of the Cardiff University (REC791; 24th May 2021).

### Participants

We used adverts in the university intranet to recruit healthy people aged ≥ 18 years old, with no known neurological, cardiovascular, or musculoskeletal conditions that would affect movement. Each participant read and signed the informed consent prior to participation in the study. All methods were performed in accordance with the relevant guidelines and regulations. Twenty-one individuals participated in the study. They had a mean age of 30.8 ± 9.0 years, a mean BMI of 25.2 ± 3.9 kg/m^2^, 14 (67%) were male, 7 (33%) were female.

### Sample size calculation

The required sample size (*n* = 16) was determined according to the recommendation for estimating sample size for reliability studies, using α = 0.05, β = 0.2, *n* = 3, *p*0 = 0.4, and *p*1 = 0.7, where α is level of significance, β is the type II error, *n* is a number of data collection sessions*, p*0 is the minimally acceptable level of reliability and *p*1 is the expected level of reliability^21^.

### Instrumentation

X4 Foot & Gait Measurement System (XSENSOR Technology Corporation, Calgary, Canada). The X4 Foot & Gait Measurement System (hereafter *the insoles*) are of varying size (European sizes 36–46 +) and are approximately 1.8 ± 0.2 mm in thickness. They have a pressure range of 7–883 kilopascals (kPa) pressure resolution of 0.007 kPa, and accuracy of ± 5% full scale. Each insole consists of 235 individual sensels with approximately 1mm gap between sensels to maximize the sensing area. They have a maximum sampling rate of 150 Hz. During the study, we collected data from the insoles at a sampling rate of 60Hz. Each sensor insole incorporates a processing unit (8 GB built-in memory, size 1.7 cm × 4 cm × 5.5 cm, 42 g each) and a wireless module that is used for data transmission and for controlling the sensor insole. The data are then wirelessly transmitted to the manufacturer’s software where it is stored. The data can then be exported to a CSV file for analysis.

PODIUM force platform (BTS Bioengineering Corp., Garbagnate Milanese MI, Italy) (hereafter the *force plates*) with four sensors with patented strain gauge architecture was used as a reference system for agreement against the insoles. The platform consists of two force plates (60 × 40 × 5.7 cm in dimensions and 28kg in weight) sampled at 1000 Hz and used to determine the three components of GRF as well as its location. Force plates are generally considered a gold-standard for COP parameters and have been used to test concurrent validity in other research^22^.

### Procedures

Upon arrival, participants wore comfortable sportswear and athletic shoes. At the start, we took anthropometric measurements including weight, height, and body segments’ lengths. Appropriately sized insoles were inserted between the subjects’ socks and the soles of the shoes. It was verified that the insoles did not fold when the subject inserted his/her foot. In the first data collection session participants performed the following activities while data from the insoles and the force plates were simultaneously being recorded: double leg squats (eight repetitions), vertical jumps (eight repetitions), and the 30-s sit-to-stand test (one repetition)^23^. Data collection from both systems were initiated shortly prior to participants commencing the trials and deemed acceptable if both feet remained in full contact with force plates through the whole duration of the trials. After this, participants performed level walking (twice 15 m, continuously in a corridor) and stair ambulation (one-floor level four times—two times up and down) while data from the insoles were only being recorded. Once all activities were completed, the researcher (TC) removed the insoles from the shoes and the participants rested for around 10 min. Then the insoles were placed by the same researcher, and participants repeated the squats (eight repetitions), vertical jumps (eight repetitions), level walking (twice 15 m, continuously in a corridor), and stair ambulation (one-floor level four times—two times up and down) while data from the insoles were only being recorded. Participants in the first data collecting session watched a demonstration by the researchers and were free to ask any questions before beginning each task. Participants engaged in every activity at their own pace.

### Systems’ synchronization

The data presented here was collected as part of a larger study^2^, where simultaneously to collecting data from the BTS force plates and the XSENSOR insoles, we were collecting data from Xsens Awinda wearable sensors. We configured the Xsens Awinda system to control start and stop of a recording by the BTS force plates, by using a trigger^24^. We did not have possibility to trigger the recording of the XSENSOR insoles in the same way. Start and end of the recording form the insoles was done manually just before the start and end of the recording from the force plates. In addition, unlike in the case for force plates where recording was done per one activity, data from the insoles was being collected for several activities continually. Thus, one data file from the insoles contained data from several activities, not one activity as in the case for force plates. To ensure synchronization between the insoles and force plates, we matched the timestamps from the two systems. Although the data files from the BTS force plates do not contain timestamps, we could use the timestamps from the Xsens Awinda sensors. First, we found the first and last timestamp from the Xsens Awinda (this indicates start and end of the activity in question). We then identified these timestamps in the XSENSOR insoles data file. Subsequently, we cropped the data from the XSENSOR insoles between these two timestamps. Finally, we up sampled the XSENSOR data to match force plates’ sampling frequency, using *resample* function in MATLAB.

### Outcomes

We collected data on vGRFs and trajectories of the COP in anterior–posterior (AP) and medial–lateral direction (ML) with the force plates, and these outcomes and their respective equivalents collected from the insoles were used to assess the agreement between the two systems. These outcomes were recorded and computed using the SMART-Capture software (BTS Bioengineering Corp., Garbagnate Milanese MI, Italy). We collected data on vGRFs, peak pressure, and trajectories of the COP in the anterior–posterior (z-axis) and the medial–lateral direction (x-axis) with the insoles, and these outcomes were used for evaluation of the test–retest reliability. These outcomes were recorded and computed using the XSENSOR X4 Foot & Gait software (XSENSOR Technology Corporation, Calgary, Canada). The definition of each outcome is presented in Table 1.

**Table 1:** Definition of the outcomes obtained from the two systems.

	The insoles		The force plates	
	Definition	Unit	Definition	Unit
vGRFs	Vertical component of the ground reaction force	N	Vertical component of the ground reaction force	N
Peak pressure	The largest pressure value in a sensor across the pressure frame	kPa	Not applicable	–
COP ML	Medial–lateral position of the centre of pressure in reference to the top left corner (right foot) or top right corner (left foot) of the pressure frame	cm	Medial–lateral position of the centre of pressure in reference to the top left corner of the force plate	cm
COP AP	Anterior–posterior position of the centre of pressure in reference to the top left corner of the pressure frame	cm	Anterior–posterior position of the centre of pressure in reference to the top left corner of the force plate	cm

COP—centre of pressure; N—Newton; PA—kilopascals; cm—centimetres.

The XSENSOR X4 Foot & Gait software reports the vGRFs as average load in pound force (lbf), thus, to allow comparison with vGRFs obtained from the force plates, it has been transformed to Newtons (N) by multiplying the values in lbf by 4.44822. Similarly, given that the XSENSOR X4 Foot & Gait software reports peak pressure in pound per square inch (PSI), we have transformed it to kPa by multiplying the values in PSI by 6.89476. The load at each sensel is estimated from the pressure at each sensel and the area of that sensel, whilst the COP is estimated by multiplying the pressure at each sensel by the coordinate of the sensel in all directions.

### Data processing

**Figure 1:**
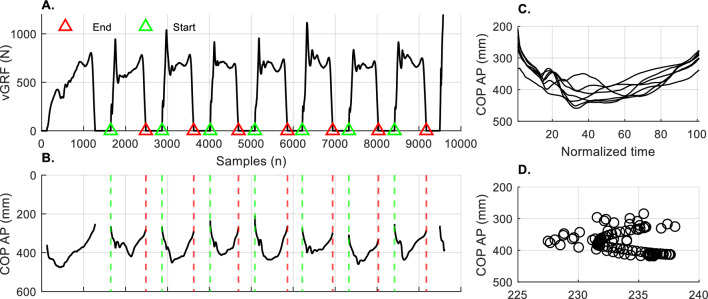
Methodological framework for data processing using a random subject’s vGRF and COP data during jumps. (**A**) Identification of the start and end of the movement cycles using vGRF data; (**B)** Segmentation of the COP data per movement cycles; (**C**) Resampling all movement cycles to 101 data points; (**D**) Scatterplot of mean trajectories of the COP; vGRFs—vertical ground reaction forces; N—Newtons; COP—Center of pressure; AP—anterior–posterior.

All data processing and statistical analyses were carried out in MATLAB (R2020b, The MathWorks Inc., Natick, MA, USA). Data collected using the insoles were exported as *.csv files and then transformed into *.mat files. Data collected using the force plates were exported as *.tdf files and then transformed into *.mat files. All raw data were filtered with a 6-Hz low-pass second-order Butterworth filter. Low-pass Butterworth digital filter was used because it is a fairly robust filter often used for biomechanical data to reduce noise and retain true signal for analysis^25^. There is no clear consensus on the ideal cut-off frequency for filtering the vGRF data, and currently available literature reports different ranges depending on the activity. Our study, included both high impact and low impact activities, and for consistency, we decided to use the 6Hz cut-off frequency. Data from the insoles and force plates were synchronized, by matching timestamps from two systems (see System’s Synchronization). Filtered data from the insoles were then up sampled to match the force plates’ sampling frequency, using the *resample* MATLAB function. For comparison purposes, we extracted data per movement cycle. Movement cycles were identified using *findpeaks* MATLAB function using the vGRFs signals from the left and right foot (Fig. 1A). For squats, the start of the movement cycle was defined as local maxima in the signal that preceded each local minima, whereas the end of the movement cycle was defined as the subsequent local maxima that succeeded each local minima in the signal. Conversely, for jumps, the sit-to-stand test, walking, and stair ambulation, the start of the movement cycle was defined as local minima in the signal that preceded each local maxima, whereas the end of the movement cycle was defined as the subsequent local minima that succeeded each local maxima in the signal (from heel strike to toe-off). After identifying the indices that defined the start and end of the movement cycle, we segmented all the signals (Fig. 1B). For data analysis purposes, we discarded the first and the last movement cycle. All movement cycles per participant were averaged and interpolated to 101 data points (Fig. 1C). We quantified the range values of the outcomes per movement cycle, defined as minimum value subtracted from maximum value (Fig. 1D). These were calculated for each participant as an average of all movement cycles, before being averaged across all participants. We divided stair ambulation into stair ascents and stair descents.

### Statistical analyses

#### Agreeement

To determine the agreement between the insoles and the force plates, we compared range values of the vGRFs, and COP trajectories in the ML and AP directions, obtained from the insoles and the force plates during squats, jumps, and the sit-to-stand test in the first data collection session. We evaluated the agreement between the two systems by calculating the mean bias between the systems and 95% limits of agreement, with the Bland–Altman analysis^26^. The Bland–Altman analysis includes a plot of the differences between two instruments against their mean. This results in the visualisation of the agreement between the two systems. The standard deviation (SD) of the differences measures the random variations around the mean, and the mean difference is the estimated bias. The presence of fixed bias, which means the results from two instruments are not comparable, is indicated if the mean value of the difference substantially deviates from 0 based on a paired-sample t-test. The Bland–Altman analysis also computes the 95% limits of agreement (mean difference ± 1.96* SD of the difference), which indicates how far apart outcomes from the systems are likely to be for most individuals^26^. The normality of the data distribution was checked with the Kolmogorov–Smirnov test^27^. If the distribution was not normal, we report median differences and 95% limits of agreement computed as median difference ± 1.45* the interquartile range (SD) of the difference.

#### Test–retest reliability

To determine the test–retest reliability of the insoles, we compared range values of the vGRFs, peak pressure and the trajectories of the COP in the ML and AP directions obtained from the insoles during squats, jumps, level walking, stair ascents, and stair descents in the first and second data collection sessions. Reliability was quantified using the Intraclass Correlation Coefficient (ICC) with the two-way random effects model (consistency), and the Standard Error of Measurement (SEM). ICC reflects relative reliability, which is the degree to which two or more sets of measures are maintained over repeated measurements^28^. ICC ≥ 0.75 indicates excellent reliability, ICC 0.4–0.74 indicates fair-to-high reliability, and ICC ≤ 0.39 indicates poor repeatability^29^. The SEM was quantified using the following equation.

$$SEM=SD x \sqrt{1}-ICC$$

SD refers to the standard deviation of the mean values of ranges from the two data collection sessions. SEM was used to evaluate absolute reliability and provides information on variability over repeated measurements^28^.

## Results

Data was not collected for four participants because the smallest size of the insoles did not fit in their shoes. We were not able to successfully synchronize data from the insoles and force plates for six participants during squats, and sit-to-stand and for two participants during jumps. This was due to broken timestamps from the Xsens Awinda system, which prevented us from identifying these in the XSENSOR data files and thus successfully synchronizing the two systems for these participants (see System’s Synchronization in the “Methods”) For this reason, we present results for the agreement between the two systems using combined data from both the left and right foot, whereas reliability results are presented separately for the left and right foot.

### Agreement

#### Bland–Altman Analysis

**Figure 2:**
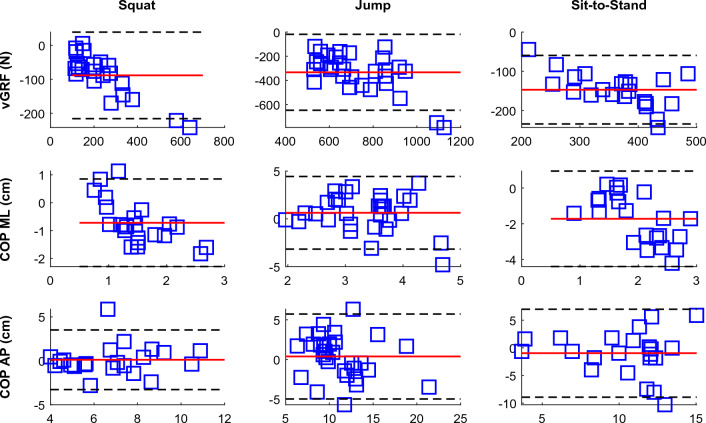
The Bland–Altman plots of the range values of the outcomes from the insoles and the force plates; y axis—differences between the two systems; x-axis—means of the two systems; red solid line—mean difference; dashed black lines—lower and upper limits of agreement (+ /− 1.96*SD of the differences); data presented for both left and right foot combined; abbreviations: vGRF—vertical ground reaction force; N—Newtons; COP—Center of pressure; ML—medial–lateral; AP—anterior–posterior.

Table 2 and Fig. 2 present the results of the Bland–Altman analysis. The results show that trajectories of the COP in anterior–posterior directions of the insoles were comparable to the corresponding COP trajectories from the force plates during all activities. However, the insoles consistently measured shorter trajectories of the COP in medial–lateral directions (except jumps) and lower values of vGRFs than the force plates, during all activities.

**Table 2:** Results of the Bland–Altman analysis comparing the range values of the outcomes collected with the insoles and the force plates during the functional activities.

Outcomes	Squat (n = 11)				Jump (n = 15)				Sit-to-Stand (n = 11)			
	Mean difference	LLA	ULA	*p*	Mean difference	LLA	ULA	*p*	Mean difference	LLA	ULA	*p*
vGRFs (N)	− 88.41	− 220.15	39.62	< 0.01	− 351.01	− 650.21	− 18.72	< 0.01	− 146.91	− 230.15	− 60.06	< 0.01
COP ML (cm)	− 0.72	− 2.34	0.85	< 0.01	0.37	− 3.21	4.43	0.05	− 1.72	− 4.47	0.95	< 0.01
COP AP (cm)	0.11	− 3.31	3.53	0.81	0.46	− 5.03	5.70	0.47	− 0.98	− 8.91	7.06	0.27

Data presented for left and right foot averaged; LLA—lower limit of agreement (− 1.96* SD of the differences); ULA—upper limit of agreement (+ 1.96* SD of the differences); vGRFs—vertical ground reaction forces; N—Newtons; COP—Center of pressure; ML—medial–lateral; AP—anterior–posterior.

#### Descriptives

**Figure 3:**
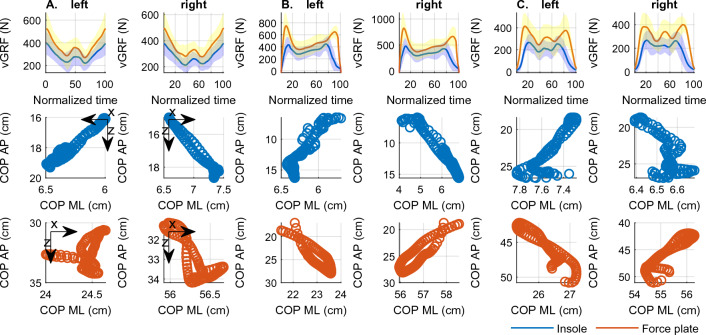
Mean (stds) participants’ waveforms for vGRF and mean scatterplots for COP trajectories for right and left foot during squats (A), jumps (B), and the sit-to-stand test (C).; abbreviations: vGRF—vertical ground reaction forces; N—Newton; COP—centre of pressure; ML—medial–lateral; AP—anterior–posterior.

Means (stds) of range values of the outcomes collected with the insoles and the force plates for both feet separately and during all activities are presented in Table 3. For descriptive purposes, Fig. 3 shows mean (stds) participants ’waveforms for vGRFs and mean scatterplots of trajectories of the COP during all activities for both feet. Individual participants ’waveforms of the outcomes for both feet and for all activities are presented in the supplementary materials (supplementary Fig. 1–3).

**Table 3:** Means [stds] of range values of the outcomes collected with the insoles and the force plates for both feet during the functional activities.

	Squat (n = 11)		Jump (n = 15)		Sit-to-Stand (n = 11)	
	Insoles	Force plates	Insoles	Force plates	Insoles	Force plates
vGRF (N)						
*L*	204.26 [128.41]	300.14 [187.35]	524.90 [122.04]	829.04 [199.53]	289.50 [63.99]	446.98 [101.58]
*R*	190.07 [107.10]	271.02 [164.19]	551.75 [139.24]	949.63 [301.24]	291.83 [62.85]	428.19 [75.14]
COP ML (cm)						
*L*	0.96 [0.37]	1.90 [0.79]	3.37 [0.91]	3.00 [1.61]	1.11 [0.62]	2.78 [1.15]
*R*	1.33 [0.34]	1.83 [0.92]	3.74 [1.43]	3.37 [1.25]	1.11 [0.43]	2.88 [1.12]
COP AP (cm)						
*L*	6.82 [2.16]	6.96 [1.95]	11.57 [3.56]	11.61 [4.44]	10.01 [3.16]	10.80 [3.80]
*R*	6.95 [ 2.42]	6.59 [2.24]	11.15 [4.05]	10.19 [4.02]	9.70 [3.19]	10.87 [3.89]

Data presented for left and right foot separately; L—left foot; R—right foot; vGRF—vertical ground reaction force; N—Newtons; COP—Center of pressure; ML—medial–lateral; AP—anterior–posterior.

### ST-retest reliability

#### ICCs

**Figure 4:**
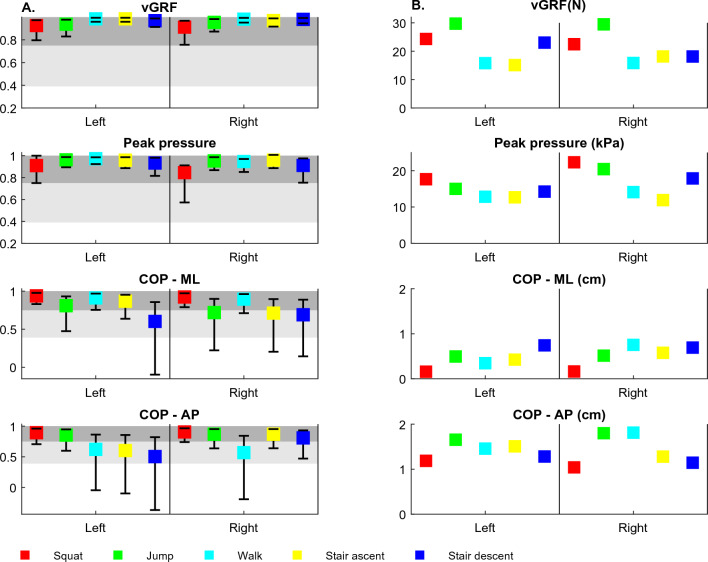
A. ICCs (95% CI) for range values of the outcomes (session 1 vs. session 2); light grey shaded areas indicate fair-to-high test–retest reliability; dark grey shaded areas indicate excellent test–retest reliability; abbreviations: vGRF—vertical ground reaction force: COP—Center of pressure; ML—medial–lateral; AP—anterior–posterior. B. SEMs for range values of the outcomes (session 1 vs. session 2); vGRF—vertical ground reaction force: COP—Center of pressure; N—Newtons; kPa—kilopascals; ML—medial–lateral; AP—anterior–posterior.

ICC values for vGRFs and peak pressure indicate excellent test–retest reliability for left and right foot during all activities. ICC values for trajectories of the COP indicate fair-to-high or excellent test–retest reliability for left and right foot during all activities (Fig. 4).

#### SEMs

SEM values were in the range of 15.1 to 29.7 N for vGRF, 11.9 to 22.3 kPa for peak pressure, 0.1 to 0.7 cm for COP in ML direction, and 1.0 to 1.8 cm for the COP in AP direction (Fig. 4).

### Descriptives

**Figure 5:**
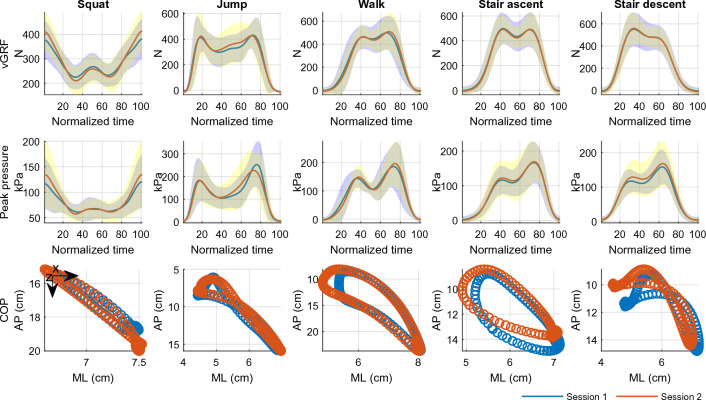
Mean (stds) participants’ waveforms for vGRFs and peak pressure and mean scatterplots for trajectories of the COP for right foot during all activities.; vGRF—vertical ground reaction force; N—Newtons; kPa—kilopascals; ML—medial–lateral; AP—anterior–posterior.

Means (stds) of range values of the outcomes collected with the insoles for all activities during the first and the second data collection sessions are presented in Table 4. For descriptive purposes, Fig. 5 shows one foot’s mean (stds) participants’ waveforms for vGRFs and peak pressure and mean scatterplots for trajectories of the COP during all activities. Individual and mean participants ’waveforms of the outcomes collected with the insoles for both feet and for all activities during the first and the second data collection sessions are presented in the supplementary materials (supplementary Figs. 4–7).

**Table 4:** Means [stds] of range values of the outcomes collected with the insoles during the first and the second data collection sessions (n = 17).

	vGRF (N)		Peak pressure (kPa)		COP ML (cm)		COP AP (cm)	
	Left foot	Right foot	Left foot	Right foot	Left foot	Right foot	Left foot	Right foot
Squat								
*S 1*	183.40 [97.94]	179.62 [78.82]	64.25 [51.16]	72.46 [49.64]	0.92 [0.60]	1.24 [0.56]	6.43 [3.24]	6.90 [3.0]
*S 2*	228.22 [81.45]	232.55 [72.59]	89.08 [66.33]	92.87 [63.50]	1.11 [0.64]	1.38 [0.77]	7.42 [4.05]	7.34 [3.71]
Jump								
*S 1*	513.85 [106.09]	545.40 [128.60]	241.38 [78.05]	296.61 [97.49]	3.25 [1.13]	3.44 [0.92]	11.66 [3.65]	10.93 [4.32]
*S 2*	549.04 [133.67]	576.53 [145.95]	255.17 [74.60]	290.40 [88.94]	3.24 [1.28]	3.57 [1.12]	11.85 [5.05]	11.38 [5.78]
Walk								
*S 1*	568.08 [132.78]	560.61 [125.62]	224.35 [72.53]	227.94 [65.09]	3.38 [1.23]	3.63 [0.91]	17.13 [2.43]	17.80 [2.07]
*S 2*	576.31 [126.86]	567.73 [114.54]	231.45 [81.43]	227.87 [56.67]	3.37 [1.10]	3.92 [0.96]	17.84 [2.34]	18.24 [2.36]
Stair ascent								
*S 1*	547.66 [130.69]	538.32 [109.78]	178.02 [69.02]	188.77 [55.64]	2.62 [1.30]	2.51 [1.18]	8.01 [2.52]	8.21 [3.95]
*S 2*	553.18 [119.30]	543.79 [100.13]	175.88 [56.19]	192.22 [62.26]	2.91 [1.12]	2.87 [1.09]	7.39 [2.33]	7.72 [3.22]
Stair descent								
*S 1*	609.22 [132.16]	599.40 [129.49]	165.33 [52.81]	162.16 [51.78]	2.93 [1.43]	3.05 [1.22]	7.40 [2.12]	7.30 [2.60]
*S 2*	603.22 [128.60]	598.33 [128.15]	166.02 [57.50]	171.05 [66.95]	2.95 [1.01]	3.12 [1.33]	6.78 [1.66]	7.44 [2.60]

S—data collection session; vGRF—vertical ground reaction; N—Newtons; kPa—kilopascals; COP—centre of pressure; ML—medial–lateral; AP—anterior–posterior.

## Discussion

The objective of the study was to compare the X4 Foot & Gait Measurement System (the insoles) with gold-standard force plates and examine the test–retest reliability of the insoles for measuring the vGRFs and the trajectories of the COP path during functional activities in healthy adults.

### Agreement

We found that the trajectories of the COP in the anterior–posterior direction (path length) measured with the insoles were comparable to the corresponding COP trajectories obtained from the force plates during all activities. However, the insoles consistently measured shorter trajectories of the COP in the medial–lateral direction (path width) (except jumps) and lower values of vGRFs than the force plates, during all activities. This indicates that the lengths of the COP trajectories are similar between the two systems during squats, jumps, and the sit-to-stand test. However, the widths of the COP trajectories and vGRFs are not comparable between the insoles and force plates during these activities.

DeBerardinis et al.^30^ observed a strong correlation between Medilogic® wireless insoles and Kistler force plates in the length of trajectories of the COP during walking in healthy adults, but a weak correlation in the width of the trajectories. Similar findings were obtained by Debbi et al.^31^ who compared the Pedar-X® insole system with the AMTI force plates during walking in healthy male adults. Stöggl et al.^32^ conducted a validation of Moticon’s OpenGo® insole system against the AMTI force plate in healthy adults during multiple functional activities. Shorter widths of the medio-lateral trajectories of the COP were obtained from the insoles, while trajectories in the anterior–posterior direction were closer to the force plates during single-leg balance and the Y-excursion test. They also observed, similar to our findings, underestimation, compared to the force plates, of the vGRFs, during jumps and walking. Conversely, Jönsson et al.^33^ showed a high correlation between the trajectories of the COP in both directions and the vGRFs obtained from the Pedar-X® insole system and the Bertec force plates during squats and leg press exercise in healthy adults. Likewise, Chesnin et al.^34^, observed good correlation and small root mean square errors for the COP trajectories obtained from the Parotec Insole System® when compared to the AMTI force plate during walking in healthy adults. Direct comparisons with other research are challenging due to the wide range of instruments employed, functional activities, outcome measurements, and statistics. However, our findings considerably contribute to the body of research by assessing a novel commercially accessible insole system during several functional tasks as well as a more difficult dynamic task: the vertical jump.

The differences between the two systems in the vGRFs can be explained by multiple reasons. The underestimation of vGRFs obtained from the insoles may be attributable to the way matrix sensors of the insoles measure vGRF compared to the force plates. Generally, insole systems measure forces perpendicular to each sensor in the matrix, hence, the force vector of each sensor is not necessarily identical, even if very close, to the vertical force vector measured with a force plate^35,36^. Sensor migration caused by shear stress at the foot-shoe interface and insole deformation could also be factors. The influence of in-shoe factors such as bending or sole thickness were not considered and may have affected the system. These mechanical processes may alter the exact detection of the vertical component of the forces on the sensors, resulting in differing vGRF readings when compared to force plate measurements. These should be taken into consideration when interpreting data collected during motions where considerable shear forces are acting on the insoles.

The validity of pressure insoles for measuring widths of the COP can be affected by sensor size, sensor arrangement, the number of sensors, individual sensor accuracy and repeatability, differences in sampling rate between the systems, and/or measurement context. Wang et al.^37^ observed that an increase in the number of sensors across the width of an insole resulted in a more accurate measurement of the width of trajectories of the COP. Therefore, a smaller number of sensors in the ML direction compared to the AP direction may be a contributing factor as well as because differences between the systems may be amplified due to the short-measured distance. Moreover, the presence of shoes in the current study may have added to the larger values of the COP in ML direction measured with the force plates. Insole systems measure trajectories of the COP (and vGRFs) at the surface between the insole and plantar sole of the foot while force plates between the sole of a shoe and the force plate.

### Test–retest reliability

In order to analyse the effectiveness of a treatment using plantar pressure analysis, it is critical to take test–retest repeatability values into account throughout several sessions. A trustworthy measuring device can reveal whether progress is taking place because the benefits of treatment may be modest in some conditions or develop gradually over time. Furthermore, precise evaluation of several functional activities is required since rehabilitation must handle complicated movement tasks. We show excellent test–retest reliability of vGRFs and peak pressure, and fair to high or excellent test–retest reliability of trajectories of the COP measured with the insoles during squats, jumps, walking and stair ambulation.

We noticed some between-foot differences in ICC values for the trajectories of the COP in AP direction during stair ambulation, which may be a consequence of slightly different placement of the insoles in the shoes or differences in insole migration throughout the duration of trials. Further, the absence of detectable differences in ICC for vGRFs and peak pressure between the feet signifies that the system can potentially be used in patients with a pathological gait to assess differences in foot pressure between the healthy and affected foot. Although direct comparisons with previous literature are difficult due to previously mentioned methodological differences between studies, current findings seem to be in accordance with previous literature investigating other similar systems in healthy adults. In the only study available to date assessing the reliability of the X4 insoles, Parker et al. demonstrated high between-day reliability and low area errors for average peak pressure and mean pressure^14^. However, the study used a calibration device, therefore authors suggested that reliability of the system should be explored in humans. Burns et al.^38^ and Renner et al.^39^ reported similar ICC values for vGRFs during flat walking measured with Loadsol® insoles. Castro et al.^40^ reported high values of ICCs for peak pressure measured with the WalkinSense® insoles during level walking. Likewise, similarly high ICC values were reported for spatio-temporal gait parameters measured during level walking with the PODOSmart® insoles and the OpenGo® insoles^41,42^.

Our findings significantly extend the current body of research by assessing the reliability of the insoles for more functional activities. Insole provides reproducible results of vGRF, peak pressures and the COP trajectories, allowing relative comparisons within and between subjects, even when the absolute values deviate from the gold standard. Future work should seek to employ reliability testing in clinical populations, such as diabetics, to assess the ability of the insoles to measure minimally clinically important difference (MCID). A threshold of 200 kPa has been suggested as a target for pressure-relieving orthotic and footwear interventions in people with diabetes and foot ulcers^43^. Methodological restrictions of our study (healthy participants, and reliability assessed on the same day) prevented us from assessing the insoles with such a clinically relevant objective.

### Practical implications

The applications of assessing foot biomechanical parameters in clinical populations are well documented. Plantar pressure analysis has advanced knowledge on foot deformity and loading during walking in people with diabetic neuropathy^44^. It has also been used to record temporal gait parameters in spinal cord injury patients^45^, and to assess the effects of specific rehabilitation exercises in people after stroke^46^. vGRFs and plantar pressure contact area were the most responsive variables in detecting changes after rehabilitation following hallux valgus surgery^47^. Having established accuracy and reliability, insole technology may provide advantages over traditional laboratory-based assessments as there is no need to walk exactly over a predetermined course to measure plantar pressures and vGRFs from each single foot independently and allow acquiring multiple steps rather than just 1–2 steps, as with force platforms. Considering a clinical situation, wireless insoles would be a better option because of their adaptability, portability, and lack of a distance restriction. Professionals will be able to reliably employ these systems to aid in the identification of illnesses, physical impairments, and other medical applications once they are aware of the precision of the measures from these systems in clinical populations.

Despite significant differences in terms of actual values measured, if we compare waveforms and scatterplots of the trajectories of the COP during all activities (Fig. 3), data patterns for the insoles and force-plates show a high qualitative correlation. This may be relevant for gait analysis, and more specifically for gait segmentation. The ability to distinguish between the key gait phases (heel strike, toe off) and use the detected events to extrapolate pertinent gait characteristics is crucial for reliably addressing a gait segmentation—and therefore a basic gait analysis.

Nonetheless, insole systems have some drawbacks that can potentially limit their practicality in a real-world scenario. While the placement of the electronic box on the lateral side of each shoe enhances comfort and prevents the user from wearing any additional belts, is generally not secured and tends to fall off (if not secured with additional tapes) during more dynamic tasks. We have previously shown that the low cost of wearable technologies or subsidization of costs by health insurance was a facilitator to employing such technologies for everyday patient use^48^. Although the financial burden varies widely between the insole technologies, most of them, including the one tested in this study, are beyond the financial capabilities of most people. Finally, along with being user-friendly, wireless insoles need to be power efficient. Research trends reveal that research on battery technology lags compared with research on other components of wearable technologies (including wireless insoles), suggesting that energy efficacy and efficiency remain an important design concern^49^.

### Strenghts and limitations

First, unlike previous similar studies in this field, we did not limit our study to the assessment of gait alone, but instead examined multiple functional activities, which revealed the potential performance of insoles in real-life setting. The second strength of this study is the comprehensive statistical analysis, which provides absolute and relative measures of validity and reliability. Moreover, the lack of standardization of footwear and walking speed increases the generalizability of results. Finally, the sample size was consistent with the original sample size calculation and also with related research studies^17,32,50^.

This study has also some limitations. First, participants were healthy adults who attended a single testing session, therefore relevance of the findings to other populations is limited. Further research is needed to assess the performance of the insoles in clinical and older populations over a multi-day testing session. Second, we did not consider the influence of in-shoe factors such as temperature and bending and variability of footwear design between participants. For example, different soles may cause a variable distance between the foot and floor during the stance phase of a movement cycle. Moreover, it is possible that increasing or decreasing the movement speeds could impact the validity of the insoles, hence future research should determine the impact of speed. We also did not isolate the individual repeatability of sensors, so further work should establish this, particularly when peak pressure is the determinant of treatment efficacy, for example, in the context of diabetes care. Finally, we did not assess the test–retest of the insoles when secured in the shoe by non-researchers. These technologies are assumed to be used and worn by patients in a real-life setting which presents certain methodological challenges. Future research could determine if wireless insoles can collect accurate and reliable biomechanical data if attached to the shoes by patients.

### Supplementary Information

Supplementary Information 1.

Supplementary Information 2.

## Data Availability

Range values of ground reaction forces and trajectories of the centre of pressure for all activities and each participant are included in this published article [and its supplementary information files]. The raw dataset analysed during the current study is available from the corresponding author on reasonable request.
