# Supplementary material for: Wireless pressure insoles for measuring ground reaction forces and trajectories of the centre of pressure during functional activities
Source: Sci Rep. 2023 Sep 11;13:14946. doi: 10.1038/s41598-023-41622-3 (PMC10495386; doi:10.1038/s41598-023-41622-3)
Supplement: Supplementary file 2 — Supplementary Information 2. [file 41598_2023_41622_MOESM2_ESM.docx]

**Title**

Wireless pressure insoles for measuring ground reaction forces and trajectories of the centre of pressure during functional activities.

**Authors**

T. Cudejko¹, K. Button², and M. Al-Amri²

**Affiliations**

¹ Department of Sport, Exercise and Rehabilitation, Northumbria University, Newcastle upon Tyne, NE1 8ST, United Kingdom

² School of Healthcare Sciences, College of Biomedical and Life Sciences, Cardiff University, Cardiff, CF14 4EP, United Kingdom

Supplementary Figure 1. Individual participants’ waveforms for vGRF, centre of pressure in x and z axes for left and right foot obtained from the force plates and the insoles during squats; abbreviations: vGRF – vertical ground reaction; N - Newton; PSI - pound per square inch; COP – centre of pressure; z – anterior-posterior; x – medial-lateral.

Supplementary Figure 2. Individual participants’ waveforms for vGRF, centre of pressure in x and z axes for left and right foot obtained from the force plates and the insoles during jumps; abbreviations: vGRF – vertical ground reaction; N - Newton; PSI - pound per square inch; COP – centre of pressure; z – anterior-posterior; x – medial-lateral.

Supplementary Figure 3. Individual participants’ waveforms for vGRF, centre of pressure in x and z axes for left and right foot obtained from the force plates and the insoles during sit-to-stand; abbreviations: vGRF – vertical ground reaction; N - Newton; PSI - pound per square inch; COP – centre of pressure; z – anterior-posterior; x – medial-lateral.

Supplementary Figure 4. Mean (stds) participants’ waveforms for vGRF and peak pressure and mean scatterplots for COP for left foot during all activities.; abbreviations: vGRF – vertical ground reaction; lbf-pound-force; PSI - pound per square inch; COP – centre of pressure; AP – anterior-posterior; ML – medial-lateral.

Supplementary Figure 5. Mean (stds) participants’ waveforms for vGRF and peak pressure and mean scatterplots for COP for right foot during all activities.; abbreviations: vGRF – vertical ground reaction; lbf-pound-force; PSI - pound per square inch; COP – centre of pressure; AP – anterior-posterior; ML – medial-lateral.

Supplementary Figure 6. Individual participants’ waveforms for load, peak pressure centre of pressure in x and z axes for left foot during all activities; abbreviations: vGRF – vertical ground reaction; lbf-pound-force; PSI - pound per square inch; COP – centre of pressure; AP – anterior-posterior; ML – medial-lateral.

Supplementary Figure 7. Individual participants’ waveforms for load, peak pressure centre of pressure in x and z axes for right foot during all activities; abbreviations: vGRF – vertical ground reaction; lbf-pound-force; PSI - pound per square inch; COP – centre of pressure; AP – anterior-posterior; ML – medial-lateral.
